# Utility of systemic staging in breast cancer patients with a positive sentinel lymph node biopsy

**DOI:** 10.1007/s11845-025-03867-x

**Published:** 2025-01-20

**Authors:** Tim Harding, Patrick James O’Donoghue, Michael Boland, Denis Evoy, Damien McCartan, Claire Rutherford, Ruth Prichard

**Affiliations:** Department of Breast Surgery, St. Vincent’s University Hospital, Dublin 4, D04 T6F4 Ireland

**Keywords:** Breast cancer, Personalised medicine, Precision oncology, Sentinel lymph node biopsy, SLNB, Staging

## Abstract

**Background:**

CT thorax, abdomen and pelvis (CT-TAP) remains the standard in the identification of metastatic disease in patients with newly diagnosed breast cancer. In patients with proven micro and macro axillary nodal metastasis, the optimal radiological technique remains controversial. A consensus on which patients with axillary nodal disease should receive radiological staging for distant disease and how this should be performed is not currently available. The aim of this study was to evaluate the yield from CT staging of the thorax, abdomen and pelvis (CT-TAP) in patients with proven nodal disease.

**Methods:**

Patients diagnosed with invasive breast cancer with a positive sentinel lymph node biopsy (SLNB) and subsequent staging CT-TAP between 2013 and 2017 were identified. Patient demographics, clinicopathological characteristics, CT-TAP findings and further imaging requirements were documented.

**Results:**

A total of 234 patients were identified. Of these, 164 (70%) were found to have macrometastasis and 70 (30%) to have micrometastasis or isolated tumour cells on SLNB. Within the macrometastasis cohort, abnormalities were noted on staging CT-TAP for 100 (61%) patients. Eighty of the 100 received follow-up assessment of abnormalities with 3 (2%) patients being diagnosed with distant metastatic disease. Within the micrometastasis group, abnormalities on CT-TAP staging were noted in 36 (52.1%) patients. Twenty-eight (40%) patients required further investigation and follow-up. No patient was found to have metastatic disease within this group.

**Conclusion:**

Patients diagnosed with micrometastatic disease of the axilla following a sentinel lymph node biopsy do not require systemic staging as it fails to detect metastatic disease.

## Introduction

Sentinel lymph node biopsy remains the gold standard of axillary assessment in clinically and radiologically node-negative patients with a new diagnosis of breast cancer [1]. Over the last decade, improved histological assessment of axillary nodes has resulted in the standardised classification of nodal positivity. This is divided into three groups—macrometastasis, micrometastasis and isolated tumour cells (ITCs). It is estimated that micrometastases (< 2 mm) or ITCs account for 10–15% of positive sentinel nodes [2]. The need for further axillary surgery as well as radiotherapy in patients who are found to have sentinel node micrometastases/ITCs has previously been investigated with evidence from a number of randomised controlled trials as well as large cohort studies demonstrating that patients with MiM/ITCs do not benefit from completion ALND or axillary radiotherapy, when outcomes including locoregional recurrence and disease-free survival are examined [3–5].

The role of radiological staging investigations to assess for distant metastatic disease in patients with sentinel node micrometastases/ITCs remains unclear. Current guidelines for the management of early, node-negative breast cancer recommend against the use of routine staging investigations [6]. The presence of nodal positivity is usually an indication for staging, most commonly with CT-TAP with the option of a radionucleotide bone scan [6–9]. The use of CT-TAP for distant staging is supported by national clinical guidelines [6, 7]; however, varying interpretation of patient selection is common. The diagnostic yield of radiological staging investigations increases with disease stage. A number of studies have found that factors including tumour size, pathological subtype (inflammatory breast cancer) and nodal positivity are significantly associated with increasing rates of distant metastases [10–12]. There remains debate as to the threshold for nodal positivity that warrants routine use of radiological staging investigations. Multiple guidelines [9, 13] advocate for routine CT staging only in patients with ≥ 3 or more positive lymph nodes. However, debate remains on whether patients with sentinel node micrometastases/ITCs actually benefit from such investigations, as the risk of incidental findings often leads to increased levels of patient anxiety, overuse of essential and valuable radiological resources and treatment delays [14].

Therefore, this study sought to evaluate the diagnostic yield of radiological staging investigations in patients found to have sentinel node micrometastasis/ITCs compared to those with macrometastatic disease.

## Methods

### Patient selection

Using a prospectively maintained histopathological database, a retrospective review was performed to identify all patients with a new diagnosis of invasive breast cancer who underwent a sentinel lymph node biopsy (SLNB) in a single specialist tertiary referral centre between the years of 2013–2017. Any patient found to have evidence of metastases within the sentinel node was recorded. Patients were excluded if they had clinical evidence of distant metastatic disease at diagnosis, if they had recurrent disease or if they had histopathological evidence of axillary disease on ultrasound-guided fine needle aspiration cytology at diagnosis. Any patient who did not have a CT-TAP or did not undergo SLNB was also excluded. Demographic data, clinicopathological characteristics as well as surgery type and hormone receptor subtype were recorded for every patient.

### SLNB assessment

All SLNB performed were localised with a single modality radioisotope involving injection of nanocolloid (Sodium Pertechnetate—99mTc). The radioisotope was injected on the day of surgery, and standard lymphoscintigraphy was performed at 1 h. SLNs were then identified intraoperatively using a gamma probe. If the operating surgeon failed to detect a signal using the probe preoperatively or if the patient was having a SLNB performed after neoadjuvant chemotherapy having been node positive at diagnosis, isosulfan blue dye was injected in a peri-areolar manner. Standard histopathological assessment of resected sentinel nodes involved serial slicing at 2-mm intervals followed by paraffin embedding. Single microtome sections were then cut at 3 lm and stained using haematoxylin and cytokeratin cam 5.2 (Clone: Cam 5.2 Becton Dickinson (BD), catalogue No: 345779). Any further levels performed were done so on selected cases, as per the dedicated breast histopathologist. All histopathological findings were reported according to the seventh edition of the TNM Classification of Malignant Tumours [15].

### Staging investigations

Radiological staging investigations were carried out in accordance with National Cancer Control Programme Guidelines [7]. Any patients with clinical stage III/IV breast cancer underwent systemic staging investigations with CT-TAP. In addition, local departmental policy also endorsed the use of systemic radiological staging for patients with any size breast malignancy with confirmed nodal metastatic disease via SLNB. Any patients due to receive neoadjuvant chemotherapy, or those found to have bilateral breast cancer, also underwent systemic staging.

### Statistical analysis

Descriptive statistics were conducted. Frequencies were calculated for continuous variables, and mean and range were used as data were normally distributed. Cross-tabulations, chi-square tests and Fisher exact tests were conducted for categorical variables to assess differences in clinicopathological characteristics and radiological outcomes between patients with micrometastatic and macrometastatic nodal disease. A *p*-value of < 0.05 was considered statistically significant. Statistical analysis was conducted using SPSS® statistical software for Windows (SPSS IBM Statistics 24).

## Results

A total of 271 patients were identified as having micro/macrometastasis on SLNB during the study period, of whom 234 met all inclusion criteria. Any patients who sought follow-up in other institutions, had previously documented breast malignancies as well as patients with clinical evidence of distant disease were excluded. One hundred and sixty-four (70%) patients who underwent SLNB were found to have macrometastatic disease, and 70 (30%) were found to have micrometastatic nodal disease or ITCs. Of the 70 patients found to have < 2 mm of nodal disease, 69 had micrometastases and 1 had ITCs.

The majority of patients were female (232/234, 99%), and the mean age at diagnosis was 57.7 years (range, 27–88 years). Of the 234 patients, 192 (82.0%) were diagnosed with invasive ductal carcinoma, 32 (13.6%) with invasive lobular carcinoma, 9 (3.8%) with mixed histology and 1 (0.4%) patient was diagnosed with a mucinous carcinoma of the breast. Regarding hormone receptor subtype, the majority of patients (185/234; 79%) had an oestrogen receptor (ER) positive tumour without human epidermal growth factor receptor (HER) 2 expression. Twenty-seven (11.5%) patients were found to be ER + HER + ; 9 (3.8%) patients were found to be ER-HER + , and 13 (5.5%) patients were found to have basal subtype (ER-HER-). Fifty-eight (24.7%) patients received neoadjuvant chemotherapy.

One hundred and twenty-eight patients underwent a wide local excision, and 106 patients underwent a mastectomy. Within the micrometastatic group, 37 (53%) patients and 33 (47%) patients underwent a wide local excision or mastectomy respectively. Similar results were seen in the macrometastasis group with 93 (56%) patients undergoing a wide local excision and 71 (44%) patients having a mastectomy.

A mean tumour size on radiological investigation (mammogram/ultrasound/MRI) of 2.5 cm (range, 0.6–8.0 cm) was seen for the entire cohort. Within the micrometastatic group, the mean radiological tumour size was 2.47 cm (range, 0.6–8.0 cm), while within the macrometastatic group, the mean radiological size was 2.51 cm (range, 0.7–7.6 cm). Lymphovascular invasion was identified in 116 patients in total, 37 (53%) patients within the micrometastatic group and 79 (48%) in the macrometastasis group. Analysis of tumour grade showed the vast majority of patients having a grade II–III tumour within both study groups. Grade II–III accounted for 64/70 (91%) of the micrometastatic group, while the same tumour grade accounted for 151/164 (92%) in the macrometastatic group. Full clinicopathological details are included in Table 1.

**Table 1:** Patient demographics

	Micrometastasis/ITC	Macrometastasis
Total	70 (30%)	164 (70%)
Age (average)	58.2	57.7
Histological subtype		
* IDC*	60 (86%)	132 (80%)
* ILC*	7 (10%)	25 (15%)
* Other*	3 (4%)	7 (5%)
Grade on core biopsy		
* I*	6 (9%)	13 (8%)
* II*	35 (50%)	93 (57%)
* III*	29 (41%)	58 (35%)
Lymphovascular invasion		
* Present*	37 (53%)	79 (48%)
* Not present*	33 (47%)	85 (52%)
Receptor status		
* ER* + */HER2-*	52 (74%)	133 (81%)
* ER* + */HER2* +	9 (13%)	18 (11%)
* ER-/HER2-*	3 (4%)	10 (6%)
* ER-/HER2* +	6 (9%)	3 (2%)
Tumour size (average)	2.47 cm	2.50 cm
Range	0.6–8.0 cm	0.7–7.6 cm

### Radiological staging

136/234 (58.1%) patients had abnormal CT-TAP findings. Within the micrometastatic group, abnormalities on CT-TAP were noted in 36/70 (52.1%) patients. The most common abnormality detected was a lung nodule(s) in 25/36 (69.5%) patients. Of these, 18/25 (72%) patients were recommended to have serial CT of the thorax, and of these, 17 were found to have stable nodules, while 1 patient proceeded to have a CT-guided biopsy of a nodule. They were subsequently diagnosed with a synchronous adenocarcinoma of the lung (final histology: PT2 N1). Seven (19.4%) patients were noted to have gynaecological findings on CT-TAP, with 6 of these having subsequent dedicated ultrasonographic examinations with no malignant findings, while one patient had an MRI pelvis showing a ‘burnt out endometrioma’. Four (11.1%) patients had an incidental finding of common bile duct dilation. Of the four patients, one patient required an MRI liver with Primovist, which found no cause for bile duct dilation, recommending a follow-up MRCP. Clinical correlation with liver function tests was recommended for the remaining three patients, with no pathological features identified. None of the patients with a final pathological nodal status of micrometastatic disease (pN1 Mi) was found to have evidence of distant metastatic disease on staging CT-TAP. Details of abnormalities seen on staging CT-TAP are shown in Table 2.

**Table 2:** Micrometastasis findings

	Abnormality on CT	Follow-up investigations	Malignant findings
Total abnormalities	36	28	0
*Lung nodules*	25	18	0
*Adnexal findings*	7	5	0
*Dilation of common bile duct*	4	4	0
Bony findings	1	1	0

Within the macrometastasis cohort, abnormalities were noted on staging CT-TAP in 100/164 (61%) patients. The most common abnormalities demonstrated on staging CT-TAP were lung nodules requiring follow-up (51 patients, 31.1%). Of these 51 patients, 39 patients had undergone a follow-up CT thorax, with the remaining 12 patients awaiting surveillance imaging of the thorax at the time of data collation. One (0.6%) patient was found to have nodules which had increased in size. A subsequent biopsy confirmed metastatic breast cancer. Eleven (6.7%) patients with macrometastatic disease had evidence of bony abnormalities on CT scan. All patients proceeded to have a radionucleotide bone scan with four (2.4%) patients ultimately requiring a bone biopsy. Of these, two patients were diagnosed with distant metastatic bone disease, while the remaining two patients were diagnosed with benign sclerotic lesions. The remaining seven patients initially diagnosed with bony abnormalities were subsequently found to have a normal radionucleotide bone scan. Sixteen (9.7%) adnexal abnormalities were shown on staging CT, of which 12 patients had a subsequent pelvic ultrasound performed. Of the 12 patients, 8 were noted to have benign adnexal cysts, 3 patients were diagnosed with uterine fibroids and 1 patient was diagnosed with adenomyosis of the uterus. No evidence of metastatic disease was found in this subgroup. Twenty-two (13.4%) patients had incidental liver findings on staging CT, the most common of which were liver cysts (11/22 patients). Eighteen patients required further radiological assessment with ultrasound or dedicated liver MRI. These did not reveal any evidence of distant metastatic disease. Overall, within the macrometastasis group, three patients were demonstrated to have evidence of distant metastatic disease.

Overall, within the total cohort, 58 patients received neoadjuvant chemotherapy, 46 (28.04%) of the macrometastasis group and 12 (17.14%) of the micrometastasis group. One patient diagnosed with lung metastasis was found to have undergone neoadjuvant chemotherapy, while the remaining two patients diagnosed with metastatic disease of the bone did not receive neoadjuvant chemotherapy.

## Discussion

This study assessed the practice of the radiological staging of breast cancer patients with nodal metastatic disease after sentinel lymph node biopsy. It confirms that routine CT-TAP is of limited benefit in patients found to have a diagnosis of sentinel node micrometastases or ITCs. No distant metastatic disease was identified in this cohort of patients, despite requiring multiple further radiological investigations in 28 patients. Within the macrometastatic cohort of patients, three were diagnosed with distant metastatic disease while investigations beyond a CT-TAP were performed in 69 patients.

The wide-spread use of modern imaging techniques has led to a drastic increase in incidental findings. The use of perioperative CT-TAP in breast cancer continues to aid in the diagnosis of distant metastasis but has also increased the number of incidentally discovered and unrelated findings. Previous studies have demonstrated that 6% of patients diagnosed with breast cancer will have evidence of metastasis at the time of diagnosis [16, 17]. As demonstrated in the findings above, 28 patients (40%) of all patients with biopsy-proven micrometastasis required diagnostic imaging following initial CT-TAP, with no patients found to have metastatic disease. These findings are mirrored in previous studies which have demonstrated an equally large number of incidental findings with only a small number of true metastases being identified, leading to further diagnostic and invasive testing, increased costs and patient anxiety [18, 19].

Incidental findings and subsequent need for further intervention have previously been described as the ‘cascade effect’ [20]. Other than the increased costs and patient anxiety, there are also the direct side effects of diagnostic imaging. The management and work-up of incidental findings place a significant radiation burden on patients. Ionising radiation is a known carcinogen with increased exposure associated with increased cancer incidence [21–23]. Per CT-TAP scan, patients are subjected to around 13 mSV or roughly 5 years of background radiation [24]. It is therefore vital to minimise patients to exposure from diagnostic imaging when sufficient evidence to do so is not clearly documented.

The identification of distant metastasis may alter patient’s treatment regimen as well as the overall prognosis [6, 25]. Multiple papers have demonstrated the utility of CT imaging for patients with node-positive disease or suspicion of distant metastasis [26]. A study by Hubbard et al. demonstrated that up to 50% of patients diagnosed with breast cancer would not meet current guideline criteria for CT imaging, resulting in a large number of potentially missed diagnoses [27]. This is in contrast to further studies showing a high false positive rate with CT imaging, especially in early breast cancers, leading to a yield of only 1% of asymptomatic synchronous metastasis [28].

Practice guidelines in the UK published by the Royal College of Radiologists differ from most current international practice [29]. In keeping with international standards, routine imaging for distant metastasis is reserved for patients with T3/T4 primary cancers, symptoms suggestive of metastatic disease, but also include patients with ≥ 4 abnormal nodes at axillary ultrasound or ≥ 4 macrometastatic nodes at axillary surgery.

The differentiation of micrometastasis and macrometastasis will have profound effects on treatment for patients (Tables 3 and 4) [30, 31]. It is widely accepted that macrometastasis, defined as tumour deposits of > 2 mm, often requires further axillary treatment such as lymph node dissection or axillary radiotherapy. Such treatment is usually not undertaken until distant metastatic disease has been outruled. The presence of isolated tumour cells or micrometastases, defined as isolated tumour cell clusters and tumour deposits from 0.2 to 2.0 mm, respectively, is treated very differently, similar to those with a negative SLNB, with many not requiring additional axillary treatment. In keeping with this differentiation, the findings of this study demonstrate that routine use of CT-TAP in patients with micrometastatic (or ITC) nodal disease should not have routine staging CT-TAP. In addition, the role of this investigation within the macrometastatic group requires further investigation as national guidelines differ. It is likely that patients with limited nodal positivity have a very low risk of distant disease and that staging investigations may only be warranted in more aggressive axillary disease as outlined in the UK Royal College of Radiologists protocol above (four nodes or more). Any algorithm for this process must limit the potential impact to the patient and healthcare system of performing unnecessary investigations with the associated radiation exposure as well as the likelihood of incidental findings.

**Table 3:** Macrometastasis findings

	Abnormality on CT	Follow-up investigations	Malignant findings
Total abnormalities	100	80	3
*Lung nodules*	51	39	1
*Bony findings*	11	11	2
*Adnexal findings*	16	12	0
*Liver findings*	22	18	0

**Table 4:** Micrometastasis findings

Metastasis on CT	Yes	No	*p*-value
Total	70 (30%)		
Age, mean (range)	59.6 (48–71.25)		
Histological subtype			0.928
* IDC*	0 (0%)	60 (100%)	
* ILC*	0 (0%)	7 (100%)	
* Other*	0 (0%)	3 (100%)	
Grade on core biopsy			0.619
* I*	0 (0%)	6 (100%)	
* II*	0 (0%)	36 (100%)	
* III*	0 (0%)	28 (100%)	
Lymphovascular invasion			1
* Present*	0 (0%)	37 (100%)	
* Not present*	0 (0%)	33 (100%)	
Receptor status			1
* ER* + */HER2-*	0 (0%)	52 (100%)	
* ER* + */HER2* +	0 (0%)	9 (100%)	
* ER-/HER2-*	0 (0%)	3 (100%)	
* ER-/HER2* +	0 (0%)	6 (100%)	
Tumour size (cm), mean (range)	2.46 (1.5–3)	1	
≤ 1.9 cm	0 (0%)	24 (100%)	
≥ 2 cm	0 (0%)	46 (100%)	

## Conclusion

The use of CT-TAP for the staging of a select group of node-positive breast cancer patients remains a valuable diagnostic tool. To date, there is little evidence to support the routine use of CT-TAP in patients with biopsy-proven ITCs or micrometastasis. Patients must undergo a significant radiation burden, with many subjected to further invasive and non-invasive testing due to the identification of incidental findings without identifying metastatic disease. This study re-enforces that staging CT-TAP is not warranted in patients with ITC and micrometastatic disease of the axilla.
